# Sex-specific association between inflammation and endothelial function relevant gene and vulnerable carotid plaque

**DOI:** 10.3389/fphys.2022.977578

**Published:** 2022-08-19

**Authors:** Jie Li, Ping Zhang, Xingyang Yi, Hua Luo, Ming Yu, Hong Chen, Chun Wang

**Affiliations:** ^1^ Department of Neurology, People’s Hospital of Deyang City, Deyang, China; ^2^ Department of Neurology, The Affiliated Hospital of Southwest Medical University, Luzhou, China; ^3^ Department of Neurology, The Suining Central Hospital, Suining, China

**Keywords:** high-risk stroke population, plaque vulnerability, inflammation, genetic polymorphism, sex

## Abstract

**Objectives:** We aimed to explore sex-specific association between genes involved in inflammation and endothelial function and vulnerable carotid plaque, a subclinical precursor of ischemic stroke.

**Methods:** Carotid plaque and plaque phenotype were assessed by carotid ultrasound in high-risk participants for stroke drawn from a multicenter, cross-sectional survey in southwestern China. We examined 18 single nucleotide polymorphisms (SNPs) in 10 genes related to inflammation and endothelial function. Sex differences in the genotype of the candidate SNPs and risk of vulnerable carotid plaques were assessed. Interaction tests were performed to identify the SNPs that might modify the association between the sex and vulnerable plaques. For SNPs with suggestive evidence for interaction with sex (*p* for interaction<0.05), stratification analysis by sex was performed to evaluate the sex-specific association between the SNP and vulnerable plaques.

**Results:** 2,644 high-risk individuals were enrolled, comprising 1,202 (45.5%) men and 1,442 (54.5%) women. Vulnerable carotid plaques were detected in 425 (16.1%) participants. Among candidate SNPs, the genotype frequencies of 5 SNPs (*TNFSF4* rs11811788, *TNFSF4* rs1234313, *IL6R* rs4845625, *VCAM1* rs2392221, and *ITGA2* rs1991013) were significantly different between sex (all *p* < 0.05). Univariable and multivariable analyses suggested that male individuals had a significantly higher prevalence of vulnerable carotid plaques (20.0% vs. 12.8%, adjusted OR 1.72, 95% CI 1.12–2.66, *p* = 0.014), while none of the candidate SNPs was significantly associated with vulnerable plaques (all *p* > 0.05). Interaction tests found the association between sex and vulnerable plaques is affected by the genotype of *IL6R* rs4845625 (*p* for interaction = 0.031). Stratification analysis revealed a strong association between *IL6R* rs4845625 and vulnerable carotid plaque in man (dominant model TT vs. CT + CC: adjusted OR 1.52, 95% CI 1.12–2.07, *p* = 0.007; codominant model TT vs. CC: adjusted OR 1.50, 95% CI 1.00–2.25, *p* = 0.048) but not in women (*p* > 0.05 in all genetic models).

**Conclusion:** The rs4845625 polymorphism in *IL6R* has sex-specific effects on vulnerable carotid plaque in Chinese Han high-risk individuals for stroke. Our findings provide a plausible genetic basis underlying the sex difference in carotid plaque vulnerability.

## Introduction

With rapidly aging population and an ongoing high prevalence of risk factors, the burden of stroke is expected to increase significantly worldwide (GBD 2016 Causes of Death Collaborators, 2017). Atherosclerosis is responsible for at least 20% of ischemic strokes, as a result of both cerebral embolism/thrombosis from an atherothrombotic plaque rupture and luminal stenosis (Petty et al., 1999; Hollander et al., 2002; Prasad, 2015). Atherosclerosis in the carotid artery can lead to plaque vulnerability, which is an important subclinical precursor of ischemic stroke and other vascular diseases (Rundek et al., 2008; Puig et al., 2020). Atherosclerosis is a diffuse, chronic inflammatory disease (Mangge and Almer, 2019; Wolf and Ley, 2019). Several different mechanisms play important roles in the pathogenesis of atherosclerosis, including endothelial injury, recruitment and activation of immune inflammatory cells, lipid accumulation, extensive degradation of extracellular matrix components, and smooth muscle cell proliferation (Berliner et al., 1995; Mangge and Almer, 2019; Wolf and Ley, 2019). Meanwhile, genetic factors play an important role in the determination of subclinical carotid atherosclerosis (Moskau et al., 2005; Zhao et al., 2008; Sacco et al., 2009). It has been reported that only 19.5% of the carotid plaque burden could be explained by the contribution of traditional vascular risk factors (Kuo et al., 2012). Therefore, genetic variants involved in endothelial function and inflammation may affect carotid atherogenesis and plaque vulnerability.

Although the incidence of ischemic stroke in young women is higher than men (especially for young adults ≤ 35 years), and several risk factors such as heart disease, heavy alcohol consumption, previous venous thromboembolism, diabetes mellitus, hypertension, migraine and use of combined oral contraceptives have been identified to contributing to these (Nightingale and Farmer, 2004; Leppert et al., 2022), the total ischemic stroke incidence rates are higher in man than in women according to the most up-to-date statistics (Tsao et al., 2022). Sex differences have also been recognized in the risk of carotid atherosclerotic plaque (Iemolo et al., 2004). Observational studies conducted in patients with moderate/severe carotid stenosis, or undergoing carotid endarterectomy reported that men had more high-risk vulnerable plaques compared with women, after controlling for cardiovascular risk factors (Hellings et al., 2007; Ota et al., 2010; Vrijenhoek et al., 2013) Although traditional cardiovascular risk factors such as age, hypertension, diabetes, and current smoking are associated with the prevalence of carotid plaque (Sturlaugsdottir et al., 2016; Bian et al., 2018; Santos-Neto et al., 2021), the variation in traditional cardiovascular risk factors between the sex could not fully explain these differences (Silander et al., 2008). Our previous study conducted in high-risk individuals for stroke also demonstrated that male individuals had a higher risk of vulnerable carotid plaque independent of classical vascular risk factors, suggesting sex-dependent genetic risk factors may play an important role in the progression of atherosclerosis (Li et al., 2021). Numerous studies have explored associations between polymorphisms in inflammation and endothelial function relevant genes and carotid atherosclerosis with few exploring the sex-specific genetic effects (Gardener et al., 2011a; Wang et al., 2011; Yi et al., 2020).

Therefore, in this study, we aimed to explore sex-dependent associations between genes involved in inflammation and endothelial function and vulnerable carotid plaque, a subclinical precursor of ischemic stroke.

## Materials and methods

### Study population

**FIGURE 1:**
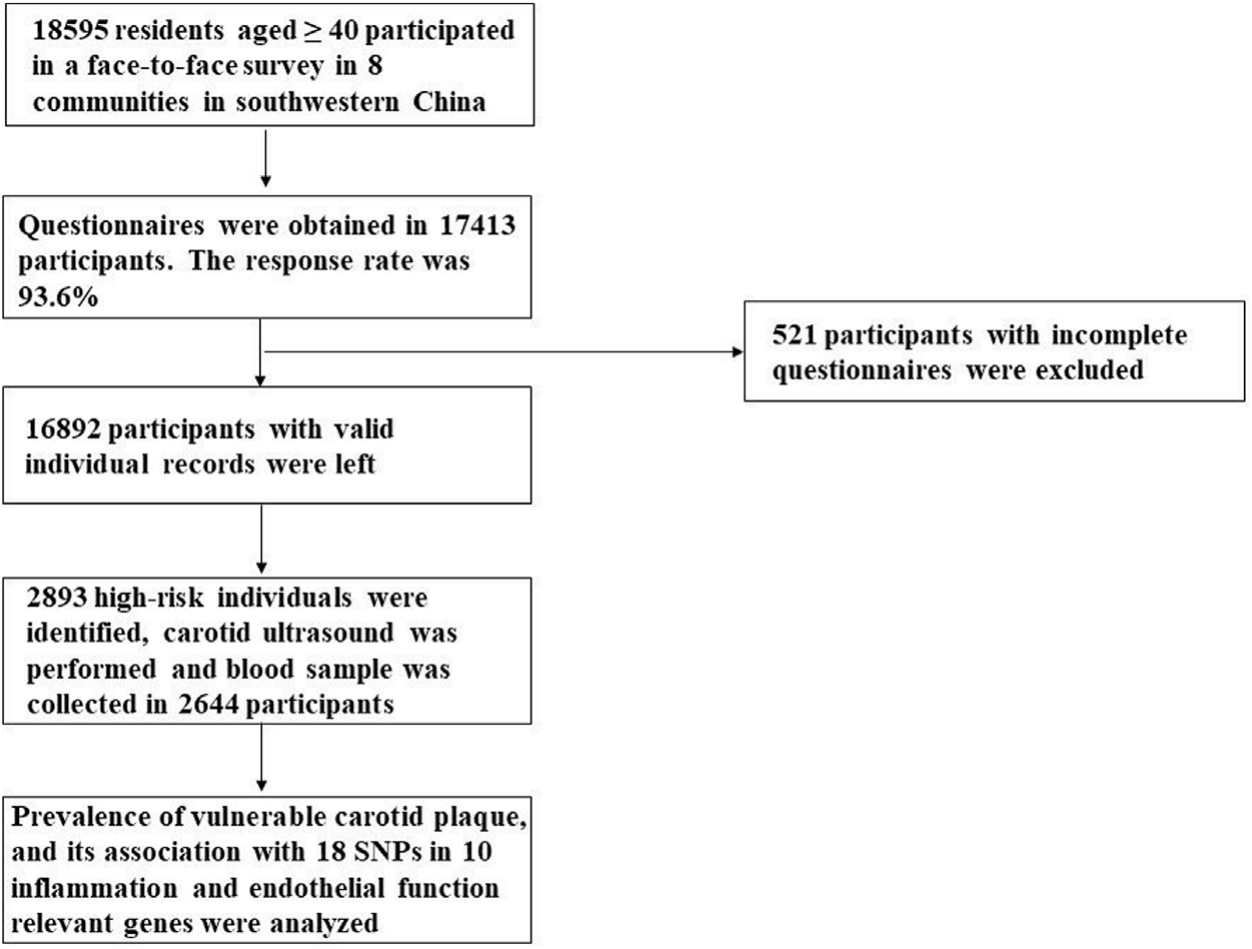
Flow diagram of the data preparing and cleaning process in this study.

18,595 residents aged ≥40 participated in a face-to-face survey in eight communities in Sichuan province in the year 2015. This multicenter, cross-sectional survey was a branch of the China National Stroke Screening Survey (CNSSS) program of the National Health and Family Planning Commission of China (grant No. 2011BAI08B01) (Li et al., 2015; Stroke Prevention Project Committee, 2022), which have been elaborated in our previous studies (Yi et al., 2020; Li et al., 2021). The eight stroke-related risk factors were evaluated, including hypertension, dyslipidemia, diabetes mellitus, atrial fibrillation, current smoking, physical inactivity, overweight/obesity and a family history of stroke, which has been described in our previous study (Yi et al., 2020; Li et al., 2021). The participants with at least three of the eight aforementioned risk factors or a history of stroke were identified as high-risk individuals for stroke (Wang et al., 2017; Stroke Prevention Project Committee, 2022). 2,644 high-risk participants for stroke who had a carotid ultrasound performed and a blood sample collected were enrolled in the present study. A flow diagram of the data preparing and cleaning process in our study is provided in Figure 1. Study protocol was approved by the Ethics Committee of the People’s Hospital of Deyang City (Reference No. 2015-024). Informed consent was obtained from each participant during recruitment.

### Carotid ultrasonography measurements

Diagnostic ultrasound was performed in 2,644 high-risk participants to assessed bilateral common and internal carotid arteries, as well as bifurcations according to a standard scanning and reading protocol (Rundek et al., 2008). Detailed procedures for evaluating the characteristics of carotid plaque have been described in our previous study (Yi et al., 2020; Li et al., 2021). Atherosclerotic plaque was defined as an endoluminal protrusion of at least 1.5 mm or a focal wall thickening>50% than the surrounding vessel wall (Rundek et al., 2008). Based on the plaque echogenicity and surface characteristics, a carotid plaque was further graded from class I to class IV as echolucent, predominantly echolucent, predominantly echogenic, and echogenic, respectively (Mathiesen et al., 2001). Carotid plaque characteristics were evaluated independently by one sonologist blinded to clinical information of participants. Plaque of class I or class II was defined as vulnerable plaque, and plaque of class III or class IV was defined as stable plaque (Yi et al., 2020). According to the results of carotid ultrasound, the enrolled participants were divided into two groups: vulnerable plaque group (with at least one vulnerable plaque) or non-vulnerable plaque group (without carotid plaque or with stable plaque).

### Gene and single nucleotide polymorphism selection

Based on a literature review (Gardener et al., 2011a; Yi et al., 2020), we selected 10 inflammation and endothelial function relevant gene that have been implicated in atherosclerosis from the NCBI database (http://www.ncbi.nlm.nih.gov/SNP). These genes included tumor necrosis factor superfamily member 4 (TNFSF4), interleukin-6 receptor (IL6R), interleukin-1α (IL1A), Toll-like receptor 4 (TLR4), tumor necrosis factor (TNF), nitric oxide synthase 2A (NOS2A), peroxisome proliferator-activated receptor-α (PPARA), vascular cell adhesion molecule-1 (VCAM-1), integrin-α2 (ITGA2), hyaluronic acid binding protein 2 (HABP2). 18 tagging or functional SNPs in these genes were evaluated.

### DNA extraction and single nucleotide polymorphism genotyping

Whole blood samples (3 ml, elbow vein) from the 2,644 participants were drawn into sterile tubes containing ethylene diamine tetraacetic acid and were stored at −80°C until genotype analysis was performed. Genomic deoxyribonucleic acid (DNA) was extracted from peripheral blood using a modified phenol/chloroform method and purified using the UNIQ-10 kit (Sangon Biotech Co., Ltd. Shanghai, China). The genotyping of the 18 SNPs was performed by investigators blinded to the basic characteristics of the participants using the matrix-assisted laser desorption/ionization time-of-flight mass spectrometry (MALDI-TOF-MS) method, which has been elaborated upon in our previous study (Yi et al., 2016). The genotype frequencies for Hardy-Weinberg equilibrium were assessed using Pearson’s chi-squared test. One SNP (*HABP2* rs7923349) which did not pass the Hardy-Weinberg equilibrium test was excluded from analyses (*p =* 6.591E-13). The remaining 17 SNPs showed no significant deviation from Hardy-Weinberg equilibrium (all *p* > 0.05).

### Statistical analyses

Sex differences in the baseline characteristics of high-risk individuals for stroke, the prevalence of carotid plaque, and genotype distributions of the 18 SNPs were assessed for significance using the χ^2^ tests (categorical variables) or the student’s *t*-tests (continuous variables). Univariable and multivariable analyses were performed to identify the risk factors associated with the prevalence of vulnerable carotid plaques. In this stage, multivariate logistic regression was performed in 2 different models. Model 1 was adjusted for variables which had a potential association with vulnerable carotid plaque in univariate analysis excluding genotype distribution which had a potentially higher risk of vulnerable plaque (*p* < 0.1), while Model 2 was adjusted for variables including genotype distribution with a potentially higher risk of vulnerable plaque (*p* < 0.1).

Then, interaction tests were performed *via* using multiple logistic regression adjusting for confounders (*p* < 0.1 in univariate models), to identify the SNPs (assuming a dominant genetic model) that might modify the association between sex and vulnerable carotid plaques. The significance of interaction was tested by the log-likelihood ratio test. A significant probability value for the interaction (*p* for interaction<0.05) would suggest that there was a sex-dependent difference in the association between the SNP and vulnerable carotid plaques.

Furthermore, stratification analysis was performed for SNPs with suggestive evidence for interaction with sex (*p* for interaction <0.05) in different genetic models (dominant, recessive and codominant). Separately in each sex stratum, multiple logistic regression was done to explore the association between each SNP and vulnerable plaques controlling for potential confounders. Sex-specific odds ratio (OR) and 95% confidence interval (CI) for SNP association were estimated in each sex stratum.

All statistical analysis was performed using SPSS v21.0 (IBM, Chicago, IL, United States), the statistical software packages R (http://www.R-project.org, The R Foundation, version 3.4.3) and EmpowerStats (http://www.empowerstats.com, X&Y Solutions, Inc., Boston, MA, United States), which have been described in our previous studies (Li et al., 2021). Two-sided *p* < 0.05 was considered to be statistically significant.

## Results

### Sex differences in the baseline characteristics of participants and prevalence of vulnerable carotid plaque

A total of 2,644 subjects at high risk of stroke were enrolled, comprising 1,202 (45.5%) men and 1,442 (54.5%) women. Carotid plaques were detected in 904 (34.2%) participants, and 479 (18.1%) subjects had stable plaques, whereas 425 (16.1%) had vulnerable plaques. Sex differences in the baseline characteristics of high-risk individuals and the prevalence of carotid plaque have been detailly described in our previous published study (Li et al., 2021). Compared with women, men were younger (62.7 ± 10.3 vs. 63.7 ± 9.4 years, *p* < 0.01), had higher levels of education (*p* < 0.01), more history of former and current smoking (13.9%, 54.2% vs. 1.7%, 4.4%, respectively, *p* < 0.01) and regular alcohol consumption (18.7% vs. 1.6%, *p* < 0.01). Meanwhile, men had larger waist circumference than women in the current survey (88.9 ± 10.0 vs. 86.4 ± 11.6 cm, *p* < 0.01). However, men had less history of ischemic stroke or transient ischemic stroke (TIA) (14.4% vs. 20.5%, *p* < 0.01), hypertension (78.6% vs. 81.6%, *p* = 0.05), diabetes (28.4% vs. 39.3%, *p* < 0.01), dyslipidemia (67.8% vs. 76.5%, *p* < 0.01) and atrial fibrillation (7.8% vs. 11.0%, *p* < 0.01) than women. The total prevalence of vulnerable carotid plaque was higher in men than in women (20.0% vs. 12.8%, *p* < 0.01).

### Sex differences in genotype distributions of the candidate single nucleotide polymorphisms

One SNP (*HABP2* rs7923349) which did not pass the Hardy-Weinberg equilibrium test was excluded from analyses (*p =* 6.591E-13). Genotype distributions of the remaining 17 SNPs were compared between sex. As shown in Table 1, the genotype frequencies of 5 SNPs (*TNFSF4* rs11811788, *TNFSF4* rs1234313, *IL6R* rs4845625, *VCAM1* rs2392221, and *ITGA2* rs1991013) were significantly different between sex (all *p* < 0.05).

**TABLE 1:** Genotype distributions of 17 SNPs in 10 genes between different sex.

Gene	SNP	Male (*n* = 1,202)	Female (*n* = 1,442)	*p* value^a^
***TNFSF4***	rs11811788(C > G)			**0.014**
	CC	972 (80.9)	1,226 (85.0)	
	CG	215 (17.9)	205 (14.2)	
	GG	15 (1.2)	11 (0.8)	
	Hardy-Weinberg *p*	0.4279	0.4530	
***TNFSF4***	rs1234313 (A > G)			**0.006**
	AA	478 (39.8)	648 (44.9)	
	AG	559 (46.5)	642 (44.5)	
	GG	165 (13.7)	152 (10.5)	
	Hardy-Weinberg *p*	0.9385	0.7065	
***IL6R***	rs1386821 (T > G)			0.727
	TT	1,116 (92.8)	1,328 (92.1)	
	GT	82 (6.8)	110 (7.6)	
	GG	4 (0.3)	4 (0.3)	
	Hardy-Weinberg *p*	0.0639	0.2872	
***IL6R***	rs4845625 (T > C)			**0.030**
	TT	366 (30.4)	373 (25.9)	
	CT	569 (47.3)	718 (49.8)	
	CC	267 (22.2)	351 (24.3)	
	Hardy-Weinberg *p*	0.1048	0.8814	
***IL1A***	rs1609682 (G > T)			0.361
	GG	593 (49.3)	674 (46.7)	
	GT	503 (41.8)	626 (43.4)	
	TT	106 (8.8)	142 (9.8)	
	Hardy-Weinberg *p*	0.9639	0.8484	
***IL1A***	rs1800587 (G > A)			0.491
	GG	1,027 (85.4)	1,255 (87.0)	
	AG	167 (13.9)	179 (12.4)	
	AA	8 (0.7)	8 (0.6)	
	Hardy-Weinberg *p*	0.6713	0.5565	
***TLR4***	rs1927911 (G > A)			0.258
	GG	458 (38.1)	505 (35.0)	
	AG	570 (47.4)	715 (49.6)	
	AA	174 (14.5)	222 (15.4)	
	Hardy-Weinberg *p*	0.8762	0.2331	
***TLR4***	rs752998 (G > T)			0.318
	GG	836 (69.6)	1,037 (71.9)	
	GT	342 (28.5)	373 (25.9)	
	TT	24 (2.0)	32 (2.2)	
	Hardy-Weinberg *p*	0.1052	0.8204	
***TNF***	rs3093662 (A > G)			0.847
	AA	1,140 (94.8)	1,370 (95.0)	
	AG	62 (5.2)	72 (5.0)	
	Hardy-Weinberg *p*	0.3587	0.3309	
***NOS2A***	rs2297518 (G > A)			0.491
	GG	867 (72.1)	1,017 (70.5)	
	AG	308 (25.6)	397 (27.5)	
	AA	27 (2.2)	28 (1.9)	
	Hardy-Weinberg *p*	0.9541	0.1318	
***NOS2A***	rs8081248 (G > A)			0.178
	GG	566 (47.1)	627 (43.5)	
	AG	518 (43.1)	665 (46.1)	
	AA	118 (9.8)	150 (10.4)	
	Hardy-Weinberg *p*	0.9739	0.1758	
***PPARA***	rs4253655 (G > A)			1.000
	GG	1,198 (99.7)	1,438 (99.7)	
	AG	4 (0.3)	4 (0.3)	
	Hardy-Weinberg *p*	0.9539	0.9579	
***PPARA***	rs4253778 (G > C)			0.740
	GG	1,197 (99.6)	1,438 (99.7)	
	CG	5 (0.4)	4 (0.3)	
	Hardy-Weinberg *p*	0.9424	0.9579	
***VCAM1***	rs2392221 (C > T)			**0.015**
	CC	864 (71.9)	1,103 (76.5)	
	CT	316 (26.3)	310 (21.5)	
	TT	22 (1.8)	29 (2.0)	
	Hardy-Weinberg *p*	0.2617	0.1915	
***ITGA2***	rs1991013 (A > G)			**0.010**
	AA	533 (44.3)	702 (48.7)	
	AG	532 (44.3)	620 (43.0)	
	GG	137 (11.4)	120 (8.3)	
	Hardy-Weinberg *p*	0.8073	0.3007	
***ITGA2***	rs4865756 (G > A)			0.279
	GG	650 (54.1)	820 (56.9)	
	AG	465 (38.7)	533 (37.0)	
	AA	87 (7.2)	89 (6.2)	
	Hardy-Weinberg *p*	0.7591	0.8474	
***HABP2***	rs932650 (T > C)			0.623
	TT	561 (46.7)	677 (46.9)	
	CT	533 (44.3)	621 (43.1)	
	CC	108 (9.0)	144 (10.0)	
	Hardy-Weinberg *p*	0.2431	0.9273	

a Determined by the chi-square test. *p* values of <0.05 are shown in bold.

### Univariable and multivariable analyses for risk factors associated with vulnerable carotid plaque

Genotype distribution of 18 SNPs between high-risk individuals with vulnerable plaque or not were presented in Table 2. Univariable analyses showed that there was a potential for differences in the genotype distributions of 3 SNPs (*TNFSF4* rs11811788, *TNFSF4* rs1234313, and *IL6R* rs4845625) between high-risk participants with vulnerable plaque or not (all *p* < 0.1). Meanwhile, as shown in our previous published study (Li et al., 2021), age, sex, family history of stroke, hypertension, smoking status, and body mass index (BMI) had a potential association with vulnerable carotid plaque (all *p* < 0.1) in univariable analyses.

**TABLE 2:** Genotype distribution between vulnerable plaque group and non-vulnerable plaque group.

Variables	Vulnerable plaque (*n* = 425)	Non-vulnerable plaque (*n* = 2,219)	*p* value^a^
*TNFSF4* (rs11811788)			**0.099**
CC	346 (81.4)	1852 (83.5)	
CG	71 (16.7)	349 (15.7)	
GG	8 (1.9)	18 (0.8)	
*TNFSF4* (rs1234313)			**0.068**
AA	160 (37.6)	966 (43.5)	
AG	213 (50.1)	988 (44.5)	
GG	52 (12.2)	265 (11.9)	
*IL6R* (rs1386821)			0.641
TT	389 (91.5)	2055 (92.6)	
GT	34 (8.0)	158 (7.1)	
GG	2 (0.5)	6 (0.3)	
*IL6R* (rs4845625)			**0.062**
TT	138 (32.5)	601 (27.1)	
CT	189 (44.5)	1,098 (49.5)	
CC	98 (23.1)	520 (23.4)	
*IL1A* (rs1609682)			0.146
GG	222 (52.2)	1,045 (47.1)	
GT	165 (38.8)	964 (43.4)	
TT	38 (8.9)	210 (9.5)	
*IL1A* (rs1800587)			0.702
GG	362 (85.2)	1920 (86.5)	
AG	61 (14.4)	285 (12.8)	
AA	2 (0.5)	14 (0.6)	
*TLR4* (rs1927911)			0.510
GG	156 (36.7)	807 (36.4)	
AG	213 (50.1)	1,072 (48.3)	
AA	56 (13.2)	340 (15.3)	
*TLR4* (rs752998)			0.709
GG	308 (72.5)	1,565 (70.5)	
GT	108 (25.4)	607 (27.4)	
TT	9 (2.1)	47 (2.1)	
*TNF* (rs3093662)			0.273
AA	408 (96.0)	2,102 (94.7)	
AG	17 (4.0)	117 (5.3)	
*NOS2A* (rs2297518)			0.998
GG	303 (71.3)	1,581 (71.2)	
AG	113 (26.6)	592 (26.7)	
AA	9 (2.1)	46 (2.1)	
NOS2A (rs8081248)			0.679
GG	200 (47.1)	993 (44.7)	
AG	183 (43.1)	1,000 (45.1)	
AA	42 (9.9)	226 (10.2)	
*PPARA* (rs4253655)			0.242
GG	422 (99.3)	2,214 (99.8)	
AG	3 (0.7)	5 (0.2)	
*PPARA* (rs4253778)			0.338
GG	422 (99.3)	2,213 (99.7)	
CG	3 (0.7)	6 (0.3)	
*VCAM1*(rs2392221)			0.201
CC	302 (71.1)	1,665 (75.0)	
CT	115 (27.1)	511 (23.0)	
TT	8 (1.9)	43 (1.9)	
*ITGA2* (rs1991013)			0.803
AA	196 (46.1)	1,039 (46.8)	
AG	184 (43.3)	968 (43.6)	
GG	45 (10.6)	212 (9.6)	
*ITGA2* (rs4865756)			0.424
GG	224 (52.7)	1,246 (56.2)	
AG	171 (40.2)	827 (37.3)	
AA	30 (7.1)	146 (6.6)	
*HABP2* (rs932650)			0.597
TT	191 (44.9)	1,047 (47.2)	
CT	195 (45.9)	959 (43.2)	
CC	39 (9.2)	213 (9.6)	

a Determined by the chi-square test. *p* values of <0.1 are shown in bold.

Multivariate logistic regression was conducted to identify the independent factors associated with the prevalence of vulnerable carotid plaques (Table 3). After adjusting for age, family history of stroke, hypertension, smoking status, and BMI (model 1), male sex was significantly associated with vulnerable carotid plaque (adjusted OR 1.11, 95% CI 1.09–1.90, *p* = 0.01). When genotype distribution of 3 SNPs (*TNFSF4* rs11811788, *TNFSF4* rs1234313, and *IL6R* rs4845625) which had a potentially higher risk of vulnerable plaque (*p* < 0.1) were included in the multivariate logistic regression (model 2), male sex was still an independent risk factor for vulnerable plaque (adjusted OR 1.43, 95% CI 1.08–1.89, *p* = 0.012). However, none of the 3 SNPs was significantly associated with vulnerable plaques (all *p* > 0.05).

**TABLE 3:** Multivariable analyses for the risk factors associated with vulnerable carotid plaque.

Variables	Multivariate analysis (model 1)	*p* value	Multivariate analysis (model 2)	*p* value
Age, yr	1.05 (1.04–1.06)	**<0.001**	1.05 (1.04–1.06)	**<0.001**
Sex (male)	1.44 (1.09–1.90)	**0.010**	1.43 (1.08–1.89)	**0.012**
Family history of stroke	0.80 (0.60–1.08)	0.141	0.80 (0.60–1.08)	0.148
Hypertension	1.44 (1.06–1.95)	**0.018**	1.45 (1.07–1.97)	**0.016**
Smoking status		**0.031**		**0.048**
Never	References	—	References	—
Former	1.31 (0.86–1.97)	0.205	1.29 (0.85–1.95)	0.234
Current	1.48 (1.11–1.99)	**0.009**	1.45 (1.08–1.95)	**0.014**
BMI	1.00 (0.97–1.03)	0.953	1.00 (0.97–1.03)	0.915
*TNFSF4* rs11811788 CC	—		1.03 (0.76–1.39)	0.848
*TNFSF4* rs1234313 AA	—		0.80 (0.63–1.01)	0.062
*IL6R* rs4845625 TT	—		1.23 (0.97–1.55)	0.082

Variables which had a potential association with vulnerable carotid plaque in univariate analysis were listed (*p* < 0.1). Figures in parentheses are 95% confidence intervals (CI).

Model 1: adjusted for variables with *p* < 0.1 in univariate analyses excluding genotype distribution which had a potentially higher risk of vulnerable plaque.

Model 2: adjusted for variables with *p* < 0.1 in univariate analyses including genotype distribution which had a potentially higher risk of vulnerable plaque.

OR, odds ratio; CI, confidence intervals; BMI, body mass index. *p* values of <0.05 are shown in bold.

Interaction tests to identify the SNP that might modify the association between sex and vulnerable carotid plaques.

We looked for evidence for interactions between the sex and SNPs in association with vulnerable carotid plaques. Among 18 SNPs in 10 genes, interaction tests revealed that only one SNP (*IL6R* rs4845625) had suggestive evidence for interaction with sex (*p* for interaction<0.05), and might modify the association between sex and vulnerable carotid plaques, as shown in Table 4.

**TABLE 4:** Interaction tests to identify the SNP that might modify the association between sex and vulnerable carotid plaque.

Variables	No. of events/total no of patients		Adjusted OR^a^ (95%CI)	*p* value^a^	*p* For interaction^a^
	Male	Female			
*TNFSF4* rs11811788					0.639
CC	190/972	156/1,226	1.36 (1.00–1.85)	0.050	
CG + GG	50/230	29/216	2.00 (1.02–3.90)	0.043	
*TNFSF4* rs1234313					0.598
AA	84/478	76/648	1.34 (0.87–2.06)	0.190	
AG + GG	156/724	109/794	1.48 (1.03–2.13)	0.033	
*IL6R* rs1386821					0.628
TT	220/1,116	169/1,328	1.40 (1.05–1.87)	0.023	
GT + GG	20/86	16/114	1.72 (0.64–4.64)	0.283	
*IL6R rs4845625*					**0.044**
TT	92/366	46/373	2.06 (1.19–3.54)	0.009	
CT + CC	148/836	139/1,069	1.27 (0.91–1.76)	0.155	
*IL1A* rs1609682					0.621
GG	125/593	97/674	1.53 (1.03–2.27)	0.033	
GT + TT	115/609	88/768	1.38 (0.93–2.04)	0.112	
*IL1A* rs1800587					0.223
GG	199/1,027	163/1,255	1.35 (1.00–1.82)	0.050	
AG + AA	41/175	22/187	1.98 (0.92–4.24)	0.079	
*TLR4* rs1927911					0.239
GG	95/458	61/505	1.62 (1.00–2.62)	0.049	
AG + AA	145/744	124/937	1.34 (0.95–1.88)	0.096	
*TLR4* rs752998					0.522
GG	174/836	134/1,037	1.48 (1.07–2.05)	0.018	
GT + TT	66/366	51/405	1.40 (0.82–2.39)	0.220	
*TNF* rs3093662					0.560
AA	229/1,140	179/1,370	1.42 (1.07–1.89)	0.016	
AG	11/62	6/72	2.03 (0.58–7.17)	0.271	
*NOS2A* rs2297518					0.454
GG	169/867	134/1,017	1.37 (0.98–1.91)	0.064	
AG + AA	71/335	51/425	1.67 (1.00–2.78)	0.048	
*NOS2A* rs8081248					0.630
GG	119/566	81/627	1.63 (1.08–2.44)	0.019	
AG + AA	121/636	104/815	1.28 (0.87–1.88)	0.204	
*PPARA* rs4253655					0.860
GG	238/1,198	184/1,438	1.41 (1.07–1.87)	0.015	
AG	2/4	1/4	0.00 (0.00, Inf)	0.995	
*PPARA* rs4253778					0.109
GG	239/1,197	183/1,438	1.44 (1.09–1.90)	0.011	
CG	1/5	2/4	0.00 (0.00, Inf)	0.994	
*VCAM1 rs2392221*					0.454
CC	168/864	134/1,103	1.63 (1.18–2.26)	0.003	
CT + TT	72/338	51/339	1.03 (0.61–1.77)	0.901	
*ITGA2 rs1991013*					0.615
AA	105/533	91/702	1.69 (1.15–2.50)	0.008	
AG + GG	135/669	94/740	1.19 (0.80–1.78)	0.382	
*ITGA2* rs4865756					0.210
GG	129/650	95/820	1.59 (1.09–2.31)	0.015	
AG + AA	111/552	90/622	1.28 (0.84–1.94)	0.255	
*HABP2* rs932650					0.668
TT	106/561	85/677	1.26 (0.83–1.92)	0.274	
CT + CC	134/641	100/765	1.59 (1.10–2.30)	0.015	

a Each stratification adjusted for age, family history of stroke, hypertension, smoking status and BMI. *p* for interaction of <0.05 are shown in bold.

### Stratification analysis for single nucleotide polymorphisms with suggestive evidence for interaction with sex

Stratification analysis was performed to explore sex-specific genotypic association of IL6R rs4845625 with vulnerable carotid plaque, in dominant, recessive and codominant model, respectively. We found that there is a strong association between *IL6R* rs4845625 and vulnerable carotid plaque in man (dominant model TT vs. CT + CC: adjusted OR 1.52, 95% CI 1.12–2.07, *p* = 0.007; codominant model TT vs. CC: adjusted OR 1.50, 95% CI 1.00–2.25, *p* = 0.048) but not in women (all *p* > 0.05), after adjusting for age, family history of stroke, hypertension, smoking status, and BMI (Table 5).

**TABLE 5:** Sex-specific genotypic association of *IL6R* rs4845625 with vulnerable carotid plaque in different genetic models.

	Model	Vulnerable carotid plaque, *n* (%)	OR (95%CI)	*p* value	Adjusted OR (95%CI)^a^	*p* value^a^
Male	Recessive			0.357		0.389
	CC (*n* = 267)	48 (18.0)	References		References	
	TT + CT (*n* = 935)	192 (20.5)	1.18 (0.83–1.67)		1.17 (0.82–1.68)	
	Dominant			**0.003**		**0.007**
	CT + CC (*n* = 836)	148 (17.7)	References		References	
	TT (*n* = 366)	92 (25.1)	1.56 (1.16–2.10)		1.52 (1.12–2.07)	
	Codominant			**0.013**		**0.027**
	CC (*n* = 267)	48 (18.0)	References	—	References	—
	CT *(n* = 569)	100 (17.6)	0.97 (0.67–1.42)	0.887	0.98 (0.67–1.45)	0.928
	TT (*n* = 366)	92 (25.1)	1.53 (1.04–2.27)	**0.033**	1.50 (1.00–2.25)	**0.048**
Female	Recessive			0.362		0.298
	CC (*n* = 351)	50 (14.2)	References		References	
	TT + CT (*n* = 1,091)	135 (12.4)	0.85 (0.60–1.21)		0.83 (0.58–1.18)	
	Dominant			0.739		0.745
	CT + CC (*n* = 1,069)	139 (13.0)	References		References	
	TT (*n* = 373)	46 (12.3)	0.94 (0.66–1.34)		0.94 (0.66–1.35)	
	Codominant			0.660		0.581
	CC (*n* = 351)	50 (14.2)	References	—	References	—
	CT (*n* = 718)	89 (12.4)	0.85 (0.59–1.24)	0.399	0.83 (0.56–1.21)	0.325
	TT (*n* = 373)	46 (12.3)	0.85 (0.55–1.30)	0.449	0.83 (0.54–1.29)	0.404

a Adjusted for age, family history of stroke, hypertension, smoking status, BMI. *p* values of <0.05 are shown in bold.

## Discussion

Although male individuals tend to have a higher risk of vulnerable carotid plaque independent of traditional vascular risk factors (Iemolo et al., 2004; Hellings et al., 2007; Ota et al., 2010; Vrijenhoek et al., 2013; Li et al., 2021), the sex-dependent genetic contribution to vulnerable plaque is still unclear. In the present study we analyzed the sex-specific genotype distribution of 10 genes involved in inflammation and endothelial function and their association with the prevalence of vulnerable carotid plaques in 2,644 high-risk individuals for stroke. Although significant difference was observed in the genotype frequencies of 5 SNPs (*TNFSF4* rs11811788, *TNFSF4* rs1234313, *IL6R* rs4845625, *VCAM1* rs2392221, and *ITGA2* rs1991013) between sex, none of the candidate SNPs was significantly associated with vulnerable carotid plaque in univariable and multivariable analyses. The sex differences in the genotype distribution of the 5 SNPs have not ever been reported in literature. However, experimental studies have shown that sex steroids might play an important role in vascular disease, *via* regulation the sex-specific expression of VCAM-1 in endothelial cells (McGrath et al., 2010; Cutini et al., 2012). The most compelling finding is that the association between sex and vulnerable plaques was affected by the genotype of *IL6R* rs4845625 in interaction tests. Further stratification analysis revealed men carrying the TT genotype of *IL6R* rs4845625 had significantly higher risk of vulnerable carotid plaque (TT vs. CT + CC: adjusted OR 1.52, 95% CI 1.12–2.07; TT vs. CC: adjusted OR 1.50, 95% CI 1.00–2.25), which was not noted in women who had a lower frequency of TT genotype than men. We have provided statistical evidence that the rs4845625 polymorphism in *IL6R* has sex-specific effects on vulnerable carotid plaque in Chinese Han high-risk individuals for stroke.

Great attention has been attracted to inflammatory molecules and their genetic variant in the pathogenesis of atherosclerosis. The human *IL6R* gene is localized on chromosome 1 band q21 (Kim et al., 2003), encoding the receptor for interleukin-6 (IL-6), which is a member of the pro-inflammatory cytokine family (Uciechowski and Dempke, 2020). IL-6 is a multifunction cytokine mainly secreted by T lymphocytes, macrophages, endothelial cells, smooth muscle cells, and adipocytes, eliciting pro-inflammatory signals in target tissues through the binding to the membrane-bound (IL6R and gp130) or circulating soluble interleukin-6 receptor (sIL6R and sgp130) on monocytes, hepatocytes, and endothelial cells (Naka et al., 2002). As we known, persistent local and systemic inflammation has been implicated in all stages of atherogenesis, from endothelial dysfunction to onset of atherosclerotic plaque rupture and their thrombotic complications (Ross, 1999), while IL-6 signaling pathway is a master player closely associated with the pathogenesis of atherosclerotic disease (Scheller and Rose-John, 2012). It has been reported that high circulating concentration of IL-6 is associated with increased risk of coronary heart disease in prospective observational studies (Ridker et al., 2000; Danesh et al., 2008). Several studies have suggested different SNPs in the *IL-6/IL-6R* were associated with several inflammatory cytokines and in relation to the susceptibility to coronary atherosclerosis (Deloukas et al., 2013; Mitrokhin et al., 2017). Meta-analyses including individual participant data from Mendelian randomization studies suggested a specific functional genetic variant Asp358Ala (rs2228145) in the *IL6R* had effects on biomarkers of inflammation and related pathways (soluble IL-6 and IL6R, C-reactive protein, fibrinogen, and others), and was associated with a reduced risk of coronary heart disease (Sarwar et al., 2012; Swerdlow et al., 2012). On the basis of genetic evidence, IL6R-related pro-inflammatory pathway seems to have a causal role in the pathogenesis of coronary atherosclerosis, and IL6R blockade could be a novel therapeutic approach for prevention of coronary atherosclerotic disease (Boekholdt and Stroes, 2012).

Compared with coronary artery disease, there is less evidence supporting IL6R signaling pathway contributing to carotid atherosclerotic diseases. Elevated IL-6 levels appear to be associated with lower echogenicity of carotid plaques, unstable plaques and internal carotid artery stenosis in several observational studies, suggesting a link between IL-6 and the pathogenesis and progression of carotid atherosclerosis (Yamagami et al., 2004; Puz et al., 2013; Hassan et al., 2020). Meanwhile, a cohort study conducted in patients undergoing carotid endarterectomy found that all components of the IL-6 signaling pathways are expressed in carotid plaques, and *IL6R* expression are higher in patients who had a history of cerebrovascular event (Ziegler et al., 2021). Thus, it is logical that the *IL-6/IL6R* gene polymorphisms could affect carotid atherosclerosis. Previous studies could not demonstrate the association between *IL-6* gene polymorphisms (which had been associated with coronary artery disease) and carotid atherosclerosis (Cunnington et al., 2009; Hulkkonen et al., 2009; Riikola et al., 2009). Until recently, there is limited information regarding the association between *IL6R* gene polymorphisms and carotid atherosclerosis. A candidate gene study examined the association between genes involved in inflammation and endothelial function carotid plaque phenotypes in the single SNP analysis, and found that *IL6R* SNP (rs1386821) was strongly associated with thick plaque phenotype (Gardener et al., 2011b). Genetic studies have indicated that the presence of the T allele of rs4845625 in the intron of the *IL6R* gene was associated with an increased risk of cardiovascular disease such as coronary artery disease and atrial fibrillation (Schnabel et al., 2011; Deloukas et al., 2013; Zhang et al., 2022). However, there is still a lack of evidence on the association between *IL6R* gene (rs4845625) polymorphism and carotid atherosclerosis. To the best of our knowledge, this is the first time we revealed that carriers of the *IL6R* rs4845625 TT genotype was associated with an increased risk of vulnerable carotid plaque in Chinese Han high-risk individuals for stroke in a sex-specific manner. Our results suggested that *IL6R* SNPs might participate in the pathogenesis of carotid atherosclerosis and plaque vulnerability in male individual. The Cardiovascular Risk in Young Finns Study also reported that the IL-6 promoter gene polymorphism (*IL6*-174 G>C) was associated with markers of subclinical carotid atherosclerosis in men, but not significant in women (Hulkkonen et al., 2009). IL6R signaling pathway could be an important therapeutic target for the prevention of carotid atherosclerosis and ischemic cerebrovascular events in male high-risk individuals. Further studies are needed to explain the molecular mechanisms in future.

Sex has long been recognized as a strong modifier of cerebrovascular disease risk. It is also worth noting that in our study population, men had more carriers of the *IL6R* rs4845625 TT genotype than women (30.4% vs. 25.9%), which had been associated with an increased risk of vulnerable carotid plaque. Sex differences have been recognized in the risk of carotid atherosclerotic plaque (Iemolo et al., 2004; Hellings et al., 2007; Ota et al., 2010; Vrijenhoek et al., 2013; Li et al., 2021). A recent prospective cohort study conducted in patients with recent ischemic cerebrovascular events and mild-to-moderate carotid stenosis also demonstrated that men are more likely to have a high-risk vulnerable carotid plaque with intraplaque hemorrhage and lipid-rich necrotic core than women, no matter the total plaque burden (van Dam-Nolen et al., 2022). Our findings provide a plausible genetic basis for the sex difference in carotid plaque vulnerability. It is not clear that how the variant rs4845625 confers sex-dependent effects on vulnerable carotid plaque. Sex-differences in other vascular risk factors could interact with the genetic variation and contribute to the sex-specific genetic effect. Future studies exploring the gene-environment interactions can help to illustrate the biological basis for the sex-specific effects.

### Limitations

The present study has several limitations. First, we only enrolled residents who were aged ≥40 years and identified as the high-risk individuals for stroke, therefore, our results can not represent the whole population. Second, the main objective of this study was to explore the association between genes involved in inflammation and endothelial function with vulnerable carotid plaque, a subclinical precursor of ischemic stroke. Thus, carotid intima thickness and carotid stenosis were not involved in our analyses. Third, this is a cross-sectional study, so the prospective prediction of the *IL6R* rs4845625 genotypes effects on the development of vulnerable carotid plaque is impossible at this stage. Besides, carotid plaque vulnerability was evaluated by ultrasound but not high-resolution magnetic resonance imaging. Fourth, the mean age of female individuals at high-risk of stroke in the present study are 63.7 years old. As we know, sex hormone levels in postmenopausal women might have an effect on atherosclerosis, however, we did not collect information about the time of menopause of women, and whether women received a hormonal treatment such as estrogen/progesterone. In addition, we conducted a candidate gene study and only a total of 18 SNPs in 10 gene were examined, further study is needed to test other inflammation and endothelial function related genes to validate the findings in our study. Furthermore, we did not explore the effect of antihypertensive drugs, statins, and antiplatelet drugs on the carotid plaque vulnerability due to a lack of data. Finally, limited to the study protocol of the CNSSS program, we could not provide information on biomarkers of inflammation such as soluble IL-6 and IL6R, C-reactive protein, fibrinogen, and others. It has been reported that sex differences exist in monocyte expression of IL-6 (O’Connor et al., 2007), the measurement of IL-6 in the participants’ serum would be an experimental technique to explore the correlation that the sex-difference in the SNP polymorphism of the *ILR6* gene contributes to the level plasma IL-6. Further studies are needed to explore this issue.

## Conclusion

Despite the above limitations, the present study provides clear evidence that the rs4845625 polymorphism in *IL6R* has sex-specific effects on vulnerable carotid plaque in Chinese Han high-risk individuals for stroke. This variant might be a genetic risk factor for vulnerable carotid plaque in Chinese male individuals. Our findings provide a plausible genetic basis underlying the sex difference in carotid plaque vulnerability. A better understanding of these sex-specific genetic effects will help identify high-risk individuals for carotid atherosclerosis and new pharmaceutical targets, as well as help to design novel strategies for the prevention and treatment of ischemic stroke.

## Data Availability

The original contributions presented in the study are included in the article/Supplementary Materials, further inquiries can be directed to the corresponding author.
